# Is the fine-tuning evidence for a multiverse?

**DOI:** 10.1007/s11229-024-04621-z

**Published:** 2024-06-18

**Authors:** Philip Goff

**Affiliations:** Department of Philosophy, Durham University, 50 Old Elvet, Durham, DH1 3HN UK

**Keywords:** Fine-tuning, Design arguments for god, Multiverse, Inverse gambler’s fallacy

## Abstract

Our best current science seems to suggest the laws of physics and the initial conditions of our universe are fine-tuned for the possibility of life. A significant number of scientists and philosophers believe that the fine-tuning is evidence for the multiverse hypothesis. This paper will focus on a much-discussed objection to the inference from the fine-tuning to the multiverse: the charge that this line of reasoning commits the inverse gambler’s fallacy. Despite the existence of a literature going back decades, this philosophical debate has made little contact with scientific discussion of fine-tuning and the multiverse, which mainly revolves around a specific form of the multiverse hypothesis rooted in eternal inflation combined with string theory. Because of this, potentially important implications from science to philosophy, and vice versa, have been left underexplored. In this paper, I will take a first step at joining up these two discussions, by arguing that attention to the eternal inflation + string theory conception of the multiverse supports the inverse gambler’s fallacy charge. It does this by supporting the idea that our universe is contingently fine-tuned, thus addressing the concern that proponents of the inverse gambler’s fallacy charge have assumed this without argument.

Our best current science seems to suggest the laws of physics and the initial conditions of our universe are fine-tuned for the possibility of life. That is to say, for life to be physically possible, certain parameters in basic physics—for example, the strength of gravity, or the mass of the electron—had to have values falling in a certain range, and that range is an incredibly narrow slice of the values those parameters might have had (Davies, 2006; Leslie, 1989, Chap. 2; Lewis & Barnes, 2016; Rees, 2000). Some philosophers deny that the fine-tuning is evidence of anything in particular, except perhaps our good fortune. But a significant number of scientists and philosophers believe that the fine-tuning is evidence for the multiverse hypothesis, the theory that our universe is just one of a very large number of universes.^1^ The basic argument is as follows. If there is just one universe, it’s incredibly improbable that the right numbers for life would have come up by chance. But if there are many universes, exemplifying different values in the relevant parameters, then the existence of a fine-tuned universe becomes much more likely.

This paper will focus on a much-discussed objection (Hacking, 1987; White, 2000/2003) to the inference from the fine-tuning to the multiverse: the charge that this line of reasoning commits the *inverse gambler’s fallacy*, which consists in inferring from an event with a remarkable outcome that there must be many other events of the same kind, most of which had less remarkable outcomes. Despite the existence of a literature going back decades, this philosophical debate has made little contact with scientific discussion of fine-tuning and the multiverse, which mainly revolves around a specific form of the multiverse hypothesis rooted in eternal inflation.^2^ Because of this, potentially important implications from science to philosophy, and vice versa, have been left underexplored. Starting from the science, it could be that attention to this particular version of the multiverse either supports or casts doubt on the charge that the move from fine-tuning to a multiverse commits the inverse gambler’s fallacy. Starting from the philosophy, it would be significant if it could be shown that a high-profile scientific argument involves fallacious reasoning.

In this paper, I will take a first step at joining up these two discussions. One way of attempting to avoid the inverse gambler’s fallacy charge, which I shall call the ‘Essentialist Response,’ involves arguing that our universe is essentially fine-tuned, or at least that proponents of the inverse gambler’s fallacy charge have failed to argue to the contrary (Holder, 2002; Juhl, 2005; Manson & Thrush, 2003; Oppy, 2006). I will argue that a certain well-known scientific theory of the multiverse, involving eternal inflation and string theory, is best interpreted as a view on which our universe is contingently fine-tuned. The upshot is that proponents of this version of the multiverse are unable to adopt the Essentialist Response.

In Sect. 1, I will outline the inverse gambler’s fallacy charge, primarily as articulated by Roger White, along the way formulating a new thought experiment which overcomes a shortcoming in White’s. In Sect. 2, I will explore four versions of the Essentialist Response to the inverse gambler’s fallacy charge. I argue that at best they establish only that *if* they can defend the thesis that our universe is essentially fine-tuned, then they can avoid the inverse gambler’s fallacy charge (at least in so far as these are arguments based on the laws/initial conditions which make our universe fine-tuned for life). In Sect. 3 will argue that, on the eternal inflation + string theory conception of the multiverse, our universe turns out *not* to be essentially fine-tuned, and thus the multiverse theorist adopting this conception of the multiverse cannot consistently press the Essentialist Response.

In this way, I hope to show how the scientific and the philosophical debates interact in such a way as to undermine the case from fine-tuning to the multiverse, at least on one prominent scientific theory of the multiverse.

## The Inverse Gambler’s fallacy charge

Fine-tuning arguments are not the only way to defend a multiverse hypothesis, but I want here to focus solely on the question of whether the laws/initial conditions which constitute the fine-tuning support the multiverse hypothesis. Hence when I refer to ‘multiverse theorists’ in what follows, I mean those who defend the multiverse, at least in part, by appeal to the laws/initial conditions which constitute the fine-tuning of our universe for life.

The inverse gambler’s fallacy charge against the multiverse theorist—from now on the ‘IGFC’—originates from Ian Hacking (1987), who applied it to John Wheeler’s ‘oscillating universe’ theory. However, Roger White (2000/2003) later gave a particularly detailed form of the objection, arguing that it applies to all forms of the multiverse and not just to the oscillating universe version. I will focus here mostly on White’s version of IGFC.

In the regular gambler’s fallacy, the gambler has had a long night of bad rolls of the dice: ‘Given that it’s unlikely that in a long series of rolls there would fail to be at least one double six,’ the gambler reasons, ‘there’s a really good chance I’ll get a double six on the next roll!’ The fallacy lies in the fact that getting a double six on the next roll is not made any more likely by the results of previous rolls. In the inverse gambler’s fallacy, the gambler walks into a casino to see another player rolling a double six: ‘Given that rolling a double six is more likely if one has rolled many times,’ the gambler reasons, ‘That player must have been playing for a while!’ Again, the error arises from the mistaken belief that rolling a six *on a particular occasion*—in this case the occasion the gambler has just witnessed—is rendered more likely by the results of previous rolls.

Hacking and White accuse the multiverse theorist of committing the same fallacy. Just as the gambler sees a remarkable roll of the dice and infers on that basis that there must have been many other, less remarkable rolls, so the multiverse theorist sees the striking fine-tuning of our universe and thus concludes that there must be many other universes with less striking numbers in their physics. And just as the gambler’s inference is unwarranted given that other rolls do not alter the odds that *the roll she witnessed* will be a double six, so the multiverse theorist’s inference is unwarranted given that the presence of other universes doesn’t make it any more likely that *this universe* will be fine-tuned.

Shortly after Hacking had first introduced the IGFC, John Leslie (1988) and P. J. McGrath (1988) (independently) accused Hacking of ignoring the selection effect introduced by the fact that we couldn’t have perceived a universe that was not fine-tuned. Whilst it is trivially true that this selection effect exists, its impact on our evidential situation is more controversial. In support of the fine-tuning argument for the multiverse, McGrath offers the following analogy:

Jane 1: Jane takes a nap at the beginning of a dice rolling session, in which an unspecified number of players will simultaneously roll a pair of dice just once, on the understanding that she will be awakened if, and only if, a double-six is rolled and not before. Upon waking she infers that there were several players rolling dice.

Initially, this case seems to mirror the selection effect we find in the fine-tuning case. In reality, given that we exist, the universe must have been fine-tuned; in the analogy, given that Jane has been woken, a double six must have been rolled. Moreover, it seems that Jane’s inference was correct, as her evidence—the fact that she has been woken and hence a double-six must have been rolled—is more likely on the assumption that there are many players than it is on the assumption that there is only one. By analogy, one might be tempted to conclude that the fact that we observe a finely tuned universe is evidence that there are many universes.

In response, White distinguishes two selection effects:

- *The mere selection effect*—If we exist, there is a fine-tuned universe.
- *The converse selection effect –* If there is a fine-tuned universe, we exist.

According to White, the real situation with respect to fine-tuning exemplifies the mere selection effect but does not exemplify the converse selection effect. That’s because, on the assumption that there is a multiverse, it could have been that the next universe down was fine-tuned rather than ours, with some other folk existing instead of us. White makes the point vivid by imagining what would have had to be the case for the converse selection effect to obtain. If we were once disembodied spirits, floating around the multiverse looking for a fine-tuned universe to slip into, *then* it would have been the case that: so long as there’s a fine-tuned universe, we’re going to exist. In the absence of something like that, argues White, we should conclude that there is no converse selection effect.

In the Jane 1 case described above, both mere and converse selection effects obtain. Given that she is woken, someone must have rolled a double six (mere selection effect); and if someone rolled a double six, she would have been woken (converse selection effect). But if White is right that this doesn’t match the real world, then, in order to properly assess our evidential situation, we’re going to need an analogy that matches the real-world situation in which the mere selection effect holds but not the converse selection effect. Thus, he introduced his own version of the Jane scenario:

Jane 2 – Jane knows that she is one of an unspecified number of sleepers each of which has a unique partner who will roll a pair of dice. Each sleeper will be awakened if and only if her partner rolls a double-six. Upon waking, Jane infers that there are several sleepers and dice rollers.

We now have a case where the mere selection effect holds (given that Jane has been woken, a double six must have been rolled) but not the converse selection effect (just because a double six has been rolled, it doesn’t follow that Jane will be woken, as it could have been someone else’s partner who rolled a double six). If White is correct that the converse selection effect doesn’t hold in the actual fine-tuning case, then Jane 2 gives a better analogy for the reality of fine-tuning than Jane 1.

Working with Jane 2 as the correct analogy, what can we infer from the evidence of fine-tuning? It seems clear that Jane’s inference in Jane 2 is incorrect. The fact that she was woken entails that *her partner* rolled a double six. But whether or not there are other sleeper-partner pairs has no bearing on the likelihood of *her partner* rolling a six. By analogy, White wants to conclude, the fine-tuned evidence gives us no reason to think there are other universes. All we know is that *our universe* is fine-tuned, and whether or not there are other universes has no bearing on the likelihood of our universe being fine-tuned. Assuming there is no converse selection effect, reflection on the above analogies seem to support the IGFC.

Whilst I haven’t read this in print, many people, including professional scientists and philosophers, have suggested to me in conversation that the Jane thought experiments are problematic because the individual of focus is ‘pre-selected’ before the dice-rolling takes place.^3^ Whether or not this alters things in a problematic way, it is clearly a disanalogy to the real-world fine-tuning situation: in the Jane scenarios, Jane pre-exists the lucky dice-rolling, whereas, in the real-world, we came into existence as a result of the event that corresponds to the lucky dice-rolling, i.e., our universe coming into existence with finely tuned parameters. This potential worry is avoided in the following thought experiment:

Jane 3: Jane is the product of IVF. One day she discovers that the doctor who performed the IVF which led to her existence was suffering from a nervous breakdown, which caused the doctor to roll dice to see whether to fertilise the egg, determining to do so only if she rolled a double six. The doctor subsequently had therapy and never did this again. Given that Jane finds herself existing, she concludes that many other doctors must have adopted this decision procedure in the case of many other potential IVFs.

This avoids the worry with the Jane 2 scenarios, as Jane does not pre-exist the dice-rolling, just as we do not pre-exist the fine-tuning. But correcting this disanalogy does not remove Jane’s error. Her evidence is that a double six was rolled to decide whether *her* conception would go ahead. How many times other doctors have done this with respect to other potential conceptions has no bearing on how likely a double six was to come up in the case of her conception. In so far as we’re exploring the evidential implications of fine-tuning, we need to focus on what Jane can infer from the fact that the right numbers came up to allow her conception to go ahead.

Very often when I have raised the Jane 3 case, collocutors have wanted to respond with a slight twist on the case:

Jane 4: Jane is the product of IVF. One day she discovers that the doctor who performed the IVF which led to her existence rolled dice an indeterminate number of times to see whether to fertilise the egg, determining to do so only if she rolled a double six. Given that Jane finds herself existing, she concludes that the doctor must have rolled the dice many times to try to get a double six.

In this case, Jane would be making a correct inference. But this final scenario fails to mirror the inference of multiverse theorists in the real-world fine-tuning case. In the Jane 4 case, Jane infers that the doctor had many rolls of the dice to determine whether *her conception* would go ahead. That corresponds to us inferring that *our universe* had multiple shots at having the values of its parameters fixed, such that it proceeded with its expansion only when the fine-tuned constants came up (we might imagine the Guardian of the Cosmos waiting to see if the fine-tuned constants come up, and then allowing the universe to proceed with its expansion). But this is not the inference multiverse theorists are making. Rather they are inferring from the fact that the right numbers came up for *our* universe to the hypothesis that there must be many *other* universes where the wrong numbers came up. This corresponds to the bad inference made in the Jane 3 case.^4^

As well as defending his position with analogies similar to those outlined above, White offers a deeper analysis of what’s going on here.^5^ The core of the issue, for White, is how we ought to construe the evidence of fine-tuning. Two possibilities suggest themselves:

- E = α is life-permitting (where α is our universe).
- E′ = Some universe is life-permitting.

White then goes on to argue that E′, but not E, raises the probability of the multiverse. He supposes that ‘we can partition the space of possible outcomes of a big bang into a finite set of equally probable configurations of initial conditions and fundamental constants: {T_1_, T_2_,…, T_n_} (think of the universes as n-sided dice, for a very large n)’ such that one of T_i_, T_1,_ is the only configuration of initial conditions and fundamental constants required for the possibility of life. For E’ to be true, there needs to be a universe instantiating T_1_, as opposed to any of the other equally possible configurations. If there is just one universe, this is incredibly improbable (1/n); the more universes there are, the more likely it is that one of them will happen to instantiate T_1_. In other words, E’ is more likely if there is multiverse than if there is only a single universe:

P(E′|M) > P(E′|~M) (where M is the hypothesis that there is a multiverse).

However, for E to be true, we need not just any old universe to instantiate T_1_, we specifically need α to instantiate T_1_. And, according to White, the number of universes distinct from α has no bearing on whether α itself will happen to instantiate T_1_. As White puts it:

The events that give rise to universes are not causally related in such a way that the outcome of one renders the outcome of another more or less probable. They are like independent rolls of a die (White, 2000: 263).

In other words:

P(E|M) = P(α instantiates T_1_|M ) = 1/n = P(α instantiates T_1_|~M ) = P(E|~M)

Assuming a standard Bayesian conception of evidence as raising of probability, we can conclude that if the evidence of fine-tuning is to be construed as E’, then it is evidence for a multiverse; but if the evidence of fine-tuning is given by E, then it is not evidence for a multiverse. If the reasoning thus far is sound, therefore, the crucial question is whether our evidence should be construed as E or E’. At this point, White (2000: 264) brings in the following principle:

Total Evidence Requirement (TER): ‘…in the confirming of hypotheses, we cannot, as a general rule, set aside a specific piece of evidence in favor of a weaker piece’ (White, 2000: 254).

White (2000, p. 264) offers the following case in support of TER:

Suppose I’m wondering why I feel sick today, and someone suggests that perhaps Adam got drunk last night. I object that I have no reason to believe this hypothesis since Adam’s drunkenness would not raise the probability of me feeling sick. But, the reply goes, it does raise the probability that someone in the room feels sick, and we know that this is true, since we know that you feel sick, so the fact that someone in the room feels sick is evidence that Adam got drunk. Clearly something is wrong with this reasoning.

What has gone wrong with this reasoning, according to White, is that TER has been disrespected: the stronger evidence—that a specific individual, namely White himself, is feeling sick—has been set aside in favour of a weaker piece of evidence—namely that someone in the room feels sick. White accuses multiverse theorists of the same mistake: setting aside a stronger piece of evidence—that a specific universe, namely α, has been fine-tuned—in favour of a weaker piece of evidence—namely that some universe has been fine-tuned.

There are a number of different objections philosophers have raised to White’s argument. Some have raised putative counterexamples to TER (Epstein, 2017; Manson & Thrush, 2003). In my view, a careful analysis of TER reveals these not to be genuine counterexamples (Draper, 2020; Draper et al., 2007). Even in the absence of a deeper theoretical analysis, the Jane analogies seem to me to make a good case for construing the evidence of fine-tuning as E rather than E’. In any case, the Essentialist Response that we will focus on in this paper does not dispute this point, and so I will take it for granted in what follows that White is correct in construing the evidence of fine-tuning as pertaining to our universe.

## The Essentialist Response

The Essentialist Response to the inverse gambler’s fallacy charge questions the following implicit assumption of Hacking and White:

*The Contingency Assumption* – The parameter values of the laws of physics and initial conditions of our universe – the strength of gravity, the mass of electrons, etc. – are contingent properties of our universe. In other words, these parameters might have had different values, and hence our universe might not have been fine-tuned.

One might wonder what kind of modality is employed in the Contingency Assumption. Fine-tuning arguments standardly employ an epistemic notion of probability. And yet the discussions below focus on the identity conditions of universes, and identity conditions—what are perhaps now more commonly referred to as ‘essences’—are standardly thought of as determining metaphysical rather than epistemic modal truths. In fact, there is no clash here. In so far as plausible claims concerning metaphysical possibility or necessity are built into the background knowledge of a given Bayesian inference, they will constrain what is epistemically possible relative to that inference. For example if I am considering the evidential implications of my social status, and I build into the background knowledge for my Bayesian reasoning (A) the actual facts about both my lineage and that of the Windsor family, and (B) the metaphysical thesis that one has one’s actual parents essentially, then (in so far as that Bayesian reasoning is concerned) there will (sadly) be no epistemically possible world in which I am Elisabeth II’s child. The truth or falsity of the Contingency Assumption has analogous implications in the Bayesian reasoning of the fine-tuning argument, as we shall see.

To the best of my knowledge, this issue was first raised by Holder (2002).^6^ In White’s formalism, the Contingency Assumption is the assumption that T_1_ (the configuration of initial conditions and fundamental constants required for the possibility of life) is a contingent property of α (our universe). Rejecting this assumption, Holder explores what happens if we ‘start from the more logical tack that our universe by definition possesses as its essential properties those represented by T_1_’ and further that T_1_ could not be instantiated more than once (Holder, 2002, pp. 305–306). In other words, Holder works from the assumption that the instantiation of T_1_ is necessary and sufficient for the existence of α. Given that all sides agree that the multiverse hypothesis raises the probability that T_1_ will be instantiated, Holder’s assumption entails that the multiverse hypothesis raises the probability that α exists. Support for the multiverse is seemingly secured.

Holder’s argument is an argument for a multiverse, but is it an argument from *fine-tuning* to a multiverse? The evidence in Holder’s argument (p. 298) is not that *our universe is fine-tuned*—for Holder, necessarily, if our universe exists, then it’s fine-tuned—but rather that *our universe exists.* Nonetheless, one might plausibly take it to require explanation why, of all the universes that might have existed, a fine-tuned universe exists rather than a non-fine-tuned universe. At least, if you thought—when hypothetically assuming the Contingency Assumption—that it requires explanation why our universe is life-permitting, then—when hypothetically assuming the negation of the Contingency Assumption—you’re probably going to think it needs explaining why a fine-tuned as opposed to a non-fine-tuned universe exists. In other words, rejecting the Contingency Assumption in itself doesn’t undermine the fine-tuning argument—at least not without further argument—it merely tweaks the explanandum (although we shall further qualify the explanandum below).^7^

The problem is that Holder’s argument against the Contingency Assumption is obscure:

By defining α as a rigid designator of our universe, White is committing a modal fallacy when he goes on to assert that α might not have been life-permitting. Indeed, instead of rigidly designating our universe, on White's treatment α might as well denote any randomly chosen universe – in fact, it would seem that this is exactly what α does represent (Holder 2002: 305).

Holder seems to be implicitly assuming that if ‘α’ is a rigid designator, it must pick out our universe is terms of some of its properties, and that the only properties that could play this role are those expressed by T_1_. Hence, thinks Holder, given that White is clearly not taking T_1_ to enter into the reference-fixing condition of ‘α’—for if it did, our universe would instantiate T_1_ by definition—White is inconsistent in asserting that ‘α’ is a rigid designator.

There are two problems with this argument. Firstly, Holder seems to be ignoring the possibility that we pick out our universe at least partly by *demonstration*: by, as it were, pointing at the physical world around us, and perhaps specifying that ‘the universe’ is everything connected in space and time to the thing pointed at.^8^ It would then be a question of metaphysics, not language, what are the essential properties of the thing thus demonstrated. Secondly, even if we do pick out the universe by description, it doesn’t follow that the description determines the essence of the referent. Perhaps we pick out α as *the nearby thing that instantiates T*_*1*_. Just as there are possible worlds in which water exists without instantiating the properties we use to pick it out in the actual world—being colourless and odourless, falling from the sky, etc.—so it could be that there are possible worlds in which our universe exists without the properties we use to pick it out in the actual world. Given these two possibilities, we certainly cannot infer from the thesis that ‘α’ is a rigid designator to the thesis that T_1_ is an essential property of α.

In a publication one year later, Manson and Thrush (2003) again raised the issue of (what I’m calling) the Contingency Assumption, this time by pointing out that there are at least some theories of the identity conditions of a universe which, if true, cast doubt on it. They consider, for example, the view that each set of possible parameters defines a cosmic essence, with the cosmic essence of our universe constituted of T_1_. On this understanding of what a universe is, Holder would be correct that the instantiation of T_1_ is necessary and sufficient for the existence of our universe, and the IGFC would fail. Crucially, however, Manson and Thrush are not arguing that the Contingency Assumption is false. Their point is rather that there is a gap in the argument of Hacking and White. This opens up a potential way of avoiding the IGFC, but not one that could be consistently adopted if one’s theory of the multiverse supports the Contingency Assumption.

Another 2 years later, a paper by Cory Juhl (2005) argues that the inverse gambler’s fallacy charge fails whether or not the Contingency Assumption is true. Juhl interprets the Contingency Assumption as the metaphysical view that our universe is essentially defined by its *haecceity*, i.e., its non-qualitative particularity or *thisness*. Presumably the thought is that if our universe is not essentially defined by its properties, then there must be some aspect of it that reflects its brute particularity—the fact that it is *this thing* as opposed to some other—and this is precisely what a haecceity is defined as.^9^

So why does the IGFC fail whether or not the Contingency Assumption is true? If, contra the Contingency Assumption, our universe is essentially defined by the parameter values of its laws and initial conditions, then the IGFC fails for the reasons discussed above. But even if, in line with the Contingency Assumption, our universe is essentially defined by its haecceity, then we still find support for the multiverse hypothesis, according to Juhl. This is because the instantiation of α ‘s haecceity is much more likely on the assumption that there are many universes than it is on the assumption that there is only one universe.

One striking thing about Juhl’s article is his acceptance that the putative support for the multiverse does not derive from fine-tuning for life. As discussed above with reference to Holder’s argument, we could take the support for the multiverse hypothesis to consist in the fact that a fine-tuned universe—as opposed to a non-fine-tuned universe—exists. Even so, Juhl claims that it is not relevant that T_1_ represents the fine-tuning of the universe for life:

…appeal to fine-tuning in particular is not required in order to yield justified inference…. Fine-tunedness for life is simply one among many equally improbable ways the laws and initial conditions might have turned out…Thus one might say that it is not fine-tuning per se that does the work in the inference. (Juhl, 2005: 344)

Juhl has a point, and it also seems to apply to Holder’s argument. For Holder, a universe is essentially defined by the values of its constants. The more universes there are, he reasons, the more likely it is that the particular values that essentially define our universe would come up. However, *whatever values came up*, the same reasoning would apply. Suppose instead of our universe there had existed a universe instantiating T_6_, where T_6_ is some range of constants not compatible with the existence of life. It would still be the case that the more universes there are, the more likely it is that T_6_ would be instantiated, and hence—by Holder’s lights—the instantiation of T_6_ would have been evidence for a multiverse.^10^ Of course, there would be nobody in the T_6_ universe to reason from this evidence, but we can still consider what hypotheses would have been supported by such evidence, and what we find seems to reveal that Holder’s argument, like Juhl’s, is not an argument from fine-tuning for life.^11^

Having said that, the evidence in Holder’s argument—and Juhl’s in so far as he rejects the Contingency Assumption—effectively concerns the laws/initial conditions which, as it happens, constitute the fine-tuning of our universe for life, as it is these that (according to Holder and Juhl in so far as he rejects the Contingency Assumption) essentially define our universe. Given this, a multiverse theorist could argue that when people make inferences from ‘fine-tuning for life’ we should think of them as making inferences from the laws/constants that *happen* to constitute fine-tuning for life. I will assume in what follows that this is how proponents of the Essentialist Response should think of the matter.

When it comes to Juhl’s argument premised on accepting the Contingency Assumption, however, there is not even an appeal to the laws/initial conditions which constitute the fine-tuning. The support instead derives solely from the multiverse hypothesis making it more likely that α’s haecceity is instantiated: given that α’s haecceity is logically and metaphysically independent of T_1_, the probability of T_1_ being instantiated has no bearing on the probability of α’s haecceity being instantiated.

This seems to make support for the multiverse too easy, in that it is not based on any empirical facts about the universe beyond its mere existence. However the universe turned out, on this view, we would have had evidence for a huge number of other universes. In my view, this should make us suspicious as to whether something has gone wrong in Juhl’s reasoning, a point we will return to shortly.

In any case, the basic argument Juhl is pushing is pressed, in a bit more detail, in Graham Oppy’s discussion of fine-tuning in his (2006) book of the following year. If we are to suppose that the parameter values of our universe are non-essential features of it, then Oppy suggests we should suppose that there are H_1_,…,H_m_ equally probable haecceities, in addition to the T_1_, T_2_,…, T_m_ possible parameter values, yielding m x n possible universes: each one corresponding to one of H_1_,…,H_m_ instantiating one of T_1_, T_2_,…, T_n_. Assuming our universe exists, the probability that it will be fine-tuned is 1/n. But for our universe to exist and be fine-tuned it first needs to exist, and for it to exist, its haecceity needs to exist. Given that there are m equally probable haecceities, on the assumption that only one universe exists, there is a 1/m chance that that universe will be α. The more universe haecceities there are, the more likely it is that α’s haecceity will be instantiated; to take the extreme case, if m non-identical universe haecceities exist, then the probability that α exists is 1.

On the other hand, if the parameter values of our physics are essential to α, then Oppy thinks we should equate the probability of α existing with the probability that its configuration of initial conditions and fundamental constants are instantiated. He justifies this via an appeal to the identity of indiscernibles to support the thesis that each universe instantiates only one of T_1_, T_2_,…, T_n_. It follows from these assumptions that, if there is only one universe, the probability that our (fine-tuned) universe exists is 1/n. The more universes there are, the more likely it becomes that our fine-tuned universe will exist; in the extreme case: if there are n universes, the probability that our (fine-tuned) universe will exist is 1.

Oppy’s position is very similar to that of Juhl. In so far as he hypothetically rejects the Contingency Assumption, the justification comes from the laws and initial conditions which constitute the fine-tuning of our universe for life—as for these laws and initial conditions to be instantiated just is for our universe to exist. And in so far as Oppy is hypothetically taking the Contingency Assumption to be true, the putative support for the multiverse is totally independent of any empirical fact concerning its laws and initial conditions.^12^ As in the case of Juhl, the conclusion that there is justification for a multiverse however empirical reality turns out (so long as there is *something*) makes me suspicious.

If their account is wrong, wherein lies the error? One big problem, I think, is Oppy’s assumption, perhaps also implicitly made by Juhl, that there is a finite number of haecceities. What could possibly limit the number of possible haecceities to a certain finite number? Presumably, this is just a simplifying assumption. Nonetheless, it is a simplifying assumption that gives the wrong result. If we replace the false assumption that there is a finite number of haecceities with the surely correct assumption that there is an infinite number of possible haecceities, the supposed support for the multiverse evaporates, as no matter how many actual universes there are, so long as the number of universes is finite, the probability that α will exist is the same: 1/∞. In other words, the postulation of a large but finite number of universes does not increase the probability that our universe exists.

It is worth noting that these concerns also apply to another way of responding to the IGFC. Whilst the authors discussed thus far take the evidence of fine-tuning to be ‘α is fine-tuned’—where ‘α’ names the universe we happen to live in—Darren Bradley (2009) has suggested in response to White that I might conditionalize on *my* observations, on the assumption that I might have lived in a universe different to α.^13^ As White has himself pointed out, this view entails that there would be significant support for a multiverse even if the universe turned out not to be fine-tuned.^14^ The point is essentially the same as that raised against Oppy above. Suppose, for the sake of discussion, most possible universes contain life. For any such possible universe containing life U, it is highly unlikely that, of all the possible individuals U might have contained, U contains *me*. Therefore, if Bradley’s set-up is correct—postulating many universes to raise the probability of *my* observations—it looks like I will need to postulate very many life-containing universes to render it probable that there is a universe with *me* in it. Moreover, the deeper problem—again revisiting the charge against Oppy—is that the set-up is flawed: no matter how great the number of universes, so long as that number is finite, the probability of one of those universes containing *me*—as opposed to any other of the infinitely many possible individuals—will remain 1/∞.

To be fair, a similar charge has been made against attempts to draw evidential support from fine-tuning (Colyvan et al., 2005; McGrew et al., 2001). If the range of possible values of a given parameter is infinite, either because there is no upper limit or because there is an infinite range of possible values between any two points on the scale, then the probability that the parameter will be fine-tuned looks to be 1/∞, no matter how broad the fine-tuned range of possible values is. If this charge succeeds, then any empirical evidence regarding how apparently narrow the fine-tuned range for any given parameter is will turn out to be irrelevant; no matter how broad the range is, so long as it’s finite, the probability of its being fine-tuned will be the same.

However, there are responses that can be made to this worry about the evidential import of fine-tuning. It is not obviously obligatory to consider the entire range of possible parameter values, if there is a narrower range that is fairly natural and non-arbitrary. Collins (2009, 4.4), for example, argues for the following two theses:

(A) it is reasonable to focus on the ‘epistemically illuminated’ range, i.e., the range of parameter values for which we can assess whether or not they are life-permitting,
(B) the epistemically illuminated range is finite, because, for example, our current physical theories apply only below certain energy levels.

We can ensure this focus by building into our background knowledge that our universe is a member of a certain finite set of possible universes: the set of all universes with physical laws and initial conditions indiscernible from P—where ‘P’ rigidly designates α’s actual physical laws and initial conditions—except for having different parameter values from the epistemically illuminated range, such that, for any possible combination of parameter values from the epistemically illuminated range, there is a universe in the set that exemplifies that possible combination. Thus, effectively, we are not asking ‘How likely is a life-sustaining universe (from all of the possible universes there might have been’)?’, but rather ‘Given that our universe is in such and such finite set of possible universes (where the set is natural and non-arbitrary), how likely is it that our universe is life-sustaining?’.^15^

As Collins notes, John Leslie has previously defended something like this approach with the following analogy:

If a tiny group of flies is surrounded by a largish fly-free wall area then whether a bullet hits a fly in the group will be very sensitive to the direction in which the firer's rifle points, even if other very different areas of the wall are thick with flies. So it is sufficient to consider a local area of possible universes, e.g., those produced by slight changes in gravity's strength …. It certainly needn't be claimed that Life and Intelligence could exist only if certain force strengths, particle masses, etc. fell within certain narrow ranges … All that need be claimed is that a lifeless universe would have resulted from fairly minor changes in the forces etc. with which we are familiar. (Leslie 1989:138–9; quoted in Collins, 2009: 4.4).

Of course, one may not be satisfied with this way of accounting for the probabilities involved in fine-tuning arguments, and it is not within the business of the paper to defend it. For our purposes, we need only note that no such strategy is available when trying to ask how likely is it that the haecceity of our universe would be instantiated. To see this, we need to ask ourselves what we intend to add to the background knowledge to narrow down the range of possible haecceity’s to some finite number. Given that we are considering the evidence that α is instantiated, the fact that α is instantiated will not feature in the background knowledge. And given that haecceities are featureless, there’s nothing qualitative we could include in the background knowledge to constrain the haecceities being considered to some finite range (in the way that Collins constrains the values of the constants to a finite range).

Could we not include in the multiverse hypothesis the proposition that α is instantiated, perhaps as part of the hypothesis that all haecceities are instantiated, thus ensuring with a probability of 1 that α exists? If we’re going to just ensure the existence of α by just asserting the existence of α in the hypothesis being supported, there’s a much more parsimonious way of doing this: just go for the single universe hypothesis *that* *α* *and no other universe exists*. Assuming we don’t explicitly include in the multiverse hypothesis that α exists, then no matter how many haecceities the multiverse hypothesis asserts to exist, so long as it’s not all of them, it will still be infinitely improbable that α exists. I conclude that this source of support for the multiverse fails, as the probability of α existing on the multiverse hypothesis and the probability of α existing on the single universe hypothesis are the same, namely 1/∞.

What about the approach of taking the evidence to be *my observations*, on the assumption that I might have existed in another possible world? If we take my existence to be essentially defined by the instantiation of my haecceity, then we return to the problem of haecceities being featureless. Suppose instead we take the Kripkean view that I am essentially defined by the sperm and egg I came from. Is there any natural and non-arbitrary way of carving up a finite of set of possible worlds in which a subset is the set of possible worlds in which the sperm and egg that actually produced me come together to make a baby, so that we can evaluate the probability of my existence? I can’t see any way of doing that, and in the absence of this, it’s hard to see how we could attach any probability to my existing other than 1/∞. Perhaps there are ways of out of this, possibly drawing on some other theory of the essence of an individual person, but the challenge seems immense.

For these reasons, I’m not convinced by the arguments of Juhl and Oppy that the mere existence of the haecceity of our universe would have support for a multiverse. But even if one is unconvinced by my argument to this effect, what we can certainly say is that this putative support for the multiverse has nothing do with laws/initial conditions which constitute the fine-tuning of our universe for life; as we have discussed, it is a form of justification that is totally independent of the empirical facts of our universe beyond the brute fact of its existence.^16^ For this reason, this form of putative support for the multiverse has nothing to do with the inferences scientists make from fine-tuning discussed in the next section. In so far as we are considering arguments from the laws/initial conditions which constitute our fine-tuning for life, all of the strategies considered above require rejecting the Contingency Assumption (or at least pressing that proponents of IGFC need to defend it).

Let’s start to bring all of this together. We have explored three ways of interpreting the essentialist response:

1. Holder: The IGFC fails because it relies on the Contingency Assumption and the Contingency Assumption is false.
2. Manson and Thrush: The IGFC, as presented by Hacking and White, is incomplete as it relies on an assumption that Hacking and White have not defended, namely the Contingency Assumption.
3. Juhl and Oppy: The IGFC fails, as we gain support for the multiverse whether or not the Contingency Assumption is true.

I have argued above for the following:

- Holder’s response fails as his argument against the Contingency Assumption is unsound,
- Juhl’s and Oppy’s responses fail, as the mere fact that α’s haecceity is instantiated does not raise the probability of the multiverse if the Contingency Assumption is true.
- Even if one doubts my justification for the second claim, in so far as these are arguments from the laws/initial conditions that constitute the fine-tuning of our universe for life—and hence can plausibly be thought of as having any connection to inferences made from ‘fine-tuning for life’—all of above strategies depend on rejecting the Contingency Assumption.

This leaves Manson and Thrush’s version of the Essentialist Response. So understood, the Essentialist Response is dependent on rejecting the Contingency Assumption (or consists of pointing out that the IFGC has not been effectively pressed in the absence of support for the Contingency Assumption). Clearly, a multiverse theorist whose own conception of the multiverse commits them to the Contingency Assumption could not consistently adopt this response to protect themselves from the IGFC. In the next section, we shall explore a well-known conception of the multiverse which does precisely this.

## The inflationary multiverse

The Contingency Assumption is a metaphysical thesis about the universe we live in. What better way to address it than to look to the theories physicists offer, and then try to draw out the metaphysical implications. Surprisingly, there is very little of this in the literature on the IGFC to arguments from fine-tuning to the multiverse.

In what follows I will take a first step in rectifying this by considering the plausibility of the Contingency Assumption in relation to one well-known and reasonably well worked-out version of the multiverse hypothesis, namely that rooted in eternal inflation and string theory.^17^ Whilst the hypothesis in question is a contingent hypothesis of natural science, I will argue that it has certain implications concerning the essential nature of universes, just as the hypothesis that water is H_2_O plausibly has implications concerning the essential nature of water.

Cosmological Inflation (Guth, 1981, 2000) is the theory that the early universe enjoyed a period of exponential expansion before moving to a slower rate of expansion. This inflationary period is posited to explain the large-scale structure of the current universe, for example, the fact that the universe is flat and that the cosmic microwave background radiation is evenly distributed. In so far as these are thought of as ‘fine-tuning’ problems, inflation may deal with them. But we are not yet talking about a multiverse.

*Eternal* inflation (Steinhardt, 1983; Vilenkin, 1983) is a form of cosmological inflation according to which the inflationary period never ends for space as a whole, although it ends for regions of space. What we think of as ‘our universe’ is in fact just a region of space in which inflation has come to an end. According to eternal inflation, there are many such non-inflationary regions, or ‘bubble universes,’ separating by exponentially inflating space. Some physicists (Guth, 2001) believe that eternal models of inflation are much more plausible than non-eternal models, as the rate at which space is expanding is likely to outstrip the rate at which the field driving inflation decays.

A crucial point that is often not explicitly made in discussions of fine-tuning is that eternal inflation alone does not account for the constants and initial conditions that constitute the fine-tuning. That’s because eternal inflation is consistent with the parameters of physics being the same in all bubble universes, and if the parameters of physics were the same in all bubble universes, we would be left with no explanation of the laws/initial conditions that constitute the fine-tuning of our universe for life. To provide such an explanation, we need not just multiple universes but multiple universes with different ‘local physics,’i.e. the values of the relevant constants are different in different universes.

This is where string theory can come in. According to string theory, the fundamental constituents of reality are not particles but one-dimensional strings. Each string is located at a single point in spacetime, housed in a high-dimensional shape in which most of the dimensions are ‘curled up.’ The facts of physics, including the parameters we are concerned with in fine-tuning discussions, are fixed by the patterns of vibrations of the strings, which are in turn determined by the high-dimensional shapes in which the strings vibrate.

In fact, there is a very big number—around 10^500^—of high-dimensional shapes which could in principle house the strings, each corresponding to a different possible universe with different kinds of particles and forces. This set of possibilia is referred to as the ‘string landscape.’ This opens up the theoretical possibility that different bubble universes might exemplify different options from the string landscape. We can call the theory that posits this theoretical possibility ‘heterogenous eternal inflation.’ Proponents of this theory speculate (Tegmark, 2014: Ch. 6) that the high energies that exist during inflation are able to mould, somewhat randomly, the high-dimensional shapes contained in different regions of spacetime; as a result, different bubbles emerge from inflation with different particles and forces.

Given that we cannot observe other universes, there is no direct empirical evidence to support moving from eternal inflation to heterogenous eternal inflation. String theory is highly speculative. But even if we accept string theory, it doesn’t immediately follow that different possibilities in the landscape are actually instantiated. It is quite coherent to suppose that eternal inflation and string theory are both true, but that each bubble universe contains exactly the same particles and forces, because each point in the entire multiverse contains the same high-dimensional shape. Call the theory expressing this possibility ‘homogenous eternal inflation.’ There is no direct empirical evidence to favour heterogenous eternal inflation over homogenous eternal inflation.

One reason some take seriously heterogenous—as opposed to homogenous—eternal inflation is because it seems to provide a nice explanation of the precise laws/initial conditions that constitute the fine-tuning of our universe for life.^18^ However, assuming White is correct that the relevant evidence is that *our universe* has these precise laws/initial conditions—which in the context of eternal inflation is understood as the evidence that *our bubble universe* is fine-tuned—the crucial question is whether or not the assumption of heterogenous eternal inflation raises the probability that our bubble universe has these precise laws/initial conditions. As I have argued previously (Goff, 2023), even if there are theoretical reasons for taking heterogenous eternal inflation seriously, if we need to construe the evidence as *our bubble universe is fine-tuned*, and our bubble universe being fine-tuned is more likely on homogenous eternal inflation than heterogenous eternal inflation, then we are forced to conclude that the evidence supports the former hypothesis over the latter.

Having outlined heterogenous eternal inflation, I turn now to the question of whether the Contingency Assumption is true on this theory. According to the Contingency Assumption, the parameters contained in the laws and initial conditions of our universe might have had different values. In the context of assuming heterogenous eternal inflation ‘our universe’ means not the whole of physical reality but our bubble universe. In other words, our question is whether or not the parameters contained in the laws that specifically describe our bubble universe, and the initial conditions of our bubble universe, might have been different. In what follows I will be hypothetically assuming heterogenous eternal inflation, in order to explore its implications, and hence will take ‘α’ to refer to our bubble universe.

Assessing this question requires forming a view as to what essentially defines a bubble universe in heterogenous eternal inflation. To be clear, in what follows I will not be laying out the views of the physicists discussed above as to what essentially defines a bubble universe. This is a metaphysical question, and as such not one physicists are in general concerned with. Rather, I will argue for what I take to be plausibly a metaphysical implication of heterogenous eternal inflation.^19^

Although Manson and Thrush do not consider eternal inflation in any detail, they do suggest that ‘with cosmogenic models whereby universes grow out of inflating “bubbles” in a pre-existing hyperspace, perhaps the bubbles can be distinguished in terms of their positions in this hyperspace’ (Manson & Thrush, 2003, p. 77). This does indeed seem to be the most plausible view about the essence of bubbles in eternal inflation. Just as it’s plausible following Kripke (1980) that I am essentially defined in terms of the sperm and egg from which I originated, so it’s plausible that our bubble universe is essentially defined in terms of the region of space which stopped inflating to create this bubble. Call heterogenous eternal inflation combined with this view about the essence of bubbles in ‘EO-HEI’ (EO for ‘essential origins’).^20^

According to EO-HEI, our bubble ends up not being essentially fine-tuned. For recall, on heterogenous enteral inflation, random processes fixed the high-dimensional shapes contained in the region of space that became our universe. Those random processes happened to result in α having fine-tuned parameters, but those random processes might easily have rendered α non-fine-tuned. And crucially, at the time those random processes were moulding the high-dimensional shapes which would ultimately determine the physics in α, how many *other* regions of space were being randomly moulded had absolutely no bearing on how likely it was that the region of space that would become α would turn out fine-tuned. Although White is not working with a specific scientific model of the multiverse, the assumptions he makes about universe formation fit perfectly with EO-HEI:

The events which give rise to universes are not causally related in such a way that the outcome of one renders the outcome of another more or less probable. They are like independent rolls of a die (White, 2000: 263).

Could we not combine heterogenous eternal inflation with the ‘cosmic essence’ view of Manson and Thrush outlined earlier? On this view (call it ‘CE-HEI’, where the ‘CE’ stands for ‘cosmic essence’), α is entirely essentially defined by its parameter-values. Call the region of space which stopped inflating to become our universe ‘the seed.’ CE-HEI entails the following two propositions:

- P1: In another possible world in which random processes had moulded the seed to have different parameter-values, a universe non-identical with α would have resulted.
- P2: Any bubble universe (actual or merely possible) with the same physics as α is identical with α.

P2 is implausible. Just because another bubble universe ends up having the same physics as ours, that clearly doesn’t make it numerically identical to our universe. Despite having the same physics, these bubbles have different locations in the multiverse, and, by Leibniz’s law, identical things cannot have different properties.^21^

What about a hybrid of EO-HEI and CE-HEI? According to H-HEI—‘H’ for ‘hybrid’—α is essentially defined *both* by its location in the multiverse *and* by its parameter-values. Hence, P1 is true but P2 is false. On EO-HEI, we can take it that the seed is identical with α: a single entity merely changes state as its high-dimensional shapes are fixed and it stops inflating. On H-HEI, however, the seed and α cannot be identical, which can be demonstrated in the following:

1. Assuming H-HEI, in any non-actual world in which the universe α* which results from the seed has different physics, α is not identical with α* (this follows from P1).
2. Given that α is not identical with α*, the seed cannot be identical with both α and α* (by the transitivity of identity).
3. Either the seed is identical with both α and α* or it is identical with neither α nor α* (it would be arbitrary to say it’s identical to one but not the other).
4. Therefore, the seed is identical with neither α nor α*.

Thus, on H-HEI, we say either that the seed ceases to exist to be replaced by α, or (more plausibly) the seed continues to exist but comes to constitute a new entity α. This is an ad hoc multiplication of entities. By far the more natural assumption is that the seed *becomes* α, as an embryo becomes, in the fullness of time, an adult. The only motivation I can see for holding otherwise is to avoid the IGFC. But this would get things the wrong way around. We should be trying to work out the most plausible view as to what essentially defines α and *then* judging whether the IGFC applies, rather than fixing the game by allowing our desire to avoid this objection to shape our view of what essentially defines α.

Let us turn now to the implications for the Essentialist Response to the IGFC. We concluded in section II that extant forms of the Essentialist Response (in defence of arguments from the laws/initial conditions that constitute the fine-tuning) are dependent on rejecting the Contingency Assumption (or consist in pointing out that the IFGC has not been effectively pressed in the absence of support for the Contingency Assumption), and that a multiverse theorist whose own conception of the multiverse commits them to the Contingency Assumption could not consistently adopt this response to protect themselves from the IGFC. I conclude those arguing from the laws/conditions that constitute the fine-tuning to heterogenous eternal inflation are unable consistently to adopt the Essentialist Response.

## Conclusion

I have not considered every response that has been raised to the inverse gambler’s fallacy charge, nor every version of the multiverse hypothesis. I have rather focused on a well-known and reasonably worked-out scientific theory of the multiverse—heterogenous eternal inflation—and considered whether proponents of this view may adopt the Essentialist Response to the inverse gambler’s fallacy charge. I hope to have shown that they cannot. Proponents of heterogenous eternal inflation should either find an alternative response to the inverse gambler’s fallacy charge or accept that their view is not supported by the laws/initial conditions that constitute the fine-tuning of our universe for life.

## Footnotes

1 Scientists defending this position include Susskind (2005), Greene (2011), Tegmark (2014); philosophers include Leslie (1989), Smart (1989), Parfit (1998), Bradley (2009). The scientists mentioned here bring empirical considerations in support of inflation into their arguments for a multiverse. I argue in Goff (2023) that the inverse gambler’s fallacy discussed in this paper infect these arguments from inflation also.

2 For attempts to account for the fine-tuning in these terms, see Susskind (2005), Greene (2011), Tegmark (2014).

3 This may be connected to Epstein’s *pre-designation requirement*. I am persuaded by Draper’s (2020) response to Epstein’s case that we don’t need this principle.

4 The considerations here do not depend on events being extended over time. The point is rather that whilst there are many rolls of the dice, they are all connected to *a single egg/universe*. We could imagine the doctor in Jane 4 rolling many pairs of dice simultaneously, determining to fertilise the egg if at least one pair comes up double six. This would be analogous to many different random processes occurring simultaneously, with the one which would make our universe fine-tuned being somehow selected to determine the constants of our universe. Again, this is certainly very different from the standard multiverse inference in that we’re not inferring to the existence of many universes. Moreover, we are left with no explanation as to why, of all the random processes that might have determined the constants of our universe, the one that would lead to them being fine-tuned was selected.

5 White gives his analysis first, and then goes on to employ some of the Jane thought experiments in discussing the selection effect. I hope my ordering is exegetically more effective.

6 I am deeply indebted to Manson (2022) for informing me of the details of the literature here.

7 In White’s setup, there is only one set of laws and initial conditions which is life-sustaining. If we drop this simplifying assumption and hold, as seems likely, that the set of laws/initial conditions which essentially define our universe is one of a range of possible sets of life-sustaining laws/initial conditions (even though significantly rarer than those which are not life-sustaining), then TER will oblige us to take our evidence not as ‘a life-sustaining universe exists’ but rather as the stronger evidence that ‘a universe essentially defined by this particular set of life-sustaining laws and conditions exists.’ This is another way of seeing the point we will explore below that these are not really arguments from fine-tuning for life*.*

8 One might worry that there is something peculiar about referring to the universe by demonstration, given that we can’t point to anything distinct from the universe. However, it’s not clear to me that this objection is correctly identifying a necessary condition for demonstrative reference: I can demonstratively refer to my own mind despite the fact that I can’t ‘inwardly point’ at anybody else’s mind. I will not here explore this concern in too much detail, given the availability of the descriptive alternative outlined above. Another worry one might have in the context of inflationary multiverse theory is that ‘everything connected in space and time to the thing pointed at’ might include the whole multiverse. To avoid this, we could define ‘the universe’ relative to its origin at the big bang.

9 There is another possibility here: that our universe is essentially defined by some properties but not by the values of its constants. I do not focus on such a possibility given that it has not been explicitly formulated, as far as I’m aware. However, it would seem to lead to the same issues discussed below. In so far as we gain support for the multiverse hypothesis on the grounds that it raises the probability that the properties that essentially define our universe are instantiated, this support would be independent of the laws/initial conditions that constitute the fine-tuning of our universe.

10 Wouldn’t this form of argument apply whatever empirical reality was like? Arguably, we’d need to be able to carve up the possibilities into a finite, non-arbitrary set, as discussed below, and it’s not clear that every possible way reality could have turned out would be compatible with this.

11 If one is concerned about whether we can evaluate evidence that would obtain in a universe in which no one exists, we could imagine instead a universe which doesn’t need to be fine-tuned for life to be possible, but which nonetheless has a set of laws and initial conditions which are one among many equally improbable sets of laws and conditions. In line with the quotation from Juhl above, this seems to be what’s at the basis of this argument for the multiverse.

12 It is worth noting that later in the chapter, Oppy concedes that the IGVC is ‘clearly right in the case in which we suppose that there is a “cosmic roll of the dice” that fixes the values of the relevant parameters at some time t in the early history of the universe’ (Oppy, 2006, p. 223). I will argue in the next section this is exactly what we do find in the version of the multiverse hypothesis most popular among scientists as a way of trying to account for the fine-tuning. However, Oppy then goes on to suggest the IGVC might be avoided if we construe the evidence of fine-tuning as ‘the fact that this cosmic fine-tuning data are presented to me for consideration.’ I find it very hard to see how this would make a difference. As Oppy later considers, the IGVC would still seem to apply, as the multiverse doesn’t raise the probability that *I*—as opposed to someone else—would be subject to a presentation of the fine-tuning data. Oppy responds to this worry by pointing out that there are *many* observers subject to a presentation of the fine-tuning data. But I can’t see how a move from the first-person singular to the first-person plural changes anything: the multiverse hypothesis doesn’t raise the probability that *we*—as opposed to some other observers—will be subject to a presentation of the fine-tuning data, any more than it raises the probability that *I* will be subject to a presentation of the fine-tuning data. Along the way, Oppy considers an analogy of someone reading about a lottery winner in a newspaper: ‘If few lottery tickets are purchased, then it is extraordinarily unlikely that readers of the newspaper report will learn that there was a winning ticket (Oppy, 2006, p. 226).’ But in this case—also in the similar golfing analogy he gives—there is plausibly a converse selection effect, as whoever won the lottery the newspaper would have printed their name.

13 I’m a little unclear as to what Bradley takes to be the evidence in his fine-tuning argument for a multiverse. In his formal presentation of the argument early in the paper (pp. 63–64), he takes the evidence to be ‘at least one universe has the right constants for life.’ This is obviously the construal of the evidence White rejects on the basis of TER; and, in any case, if I take this to be the evidence, it doesn’t matter whether or not I could have existed in another universe, as the multiverse hypothesis raises the probability of this evidence whether or not I could have existed in another universe. In responding to White later, Bradley agrees with White, at least for the sake of discussion, that the multiverse hypothesis needs to raise the probability of my existence (i.e. the existence of whoever is doing the Bayesian reasoning), although without being totally clear about what proposition he’s taking to be the evidence. A central claim of the paper is that ‘we need to conditionalize on “I have learned E by process p,” as opposed to just “E”’ (p. 63), but in his formal presentation of the argument the process by which we learnt the evidence does not explicitly feature in the evidence. In personal correspondence, Bradley suggested to me that we can take the manner in which we learned the evidence as background information. But, again, if the evidence is ‘at least one universe has the right constants for life,’ then the multiverse hypothesis raises the probability of this evidence whether or not the background information contains the manner in which I discovered the evidence, and therefore this information about the manner in which the evidence was uncovered seems redundant. Bradley also suggested to me in personal correspondence that we might take the evidence to include the manner in which we discovered the evidence. But so long as my existence is included in the evidence, we are going to end up with the difficulties I outline in the main text.

14 See White’s response to objection 2 in the postscript of the reprint of his 2000/2003.

15 We can frame this in line with the rejection of the Contingency Assumption by asking: Given that what exists is in such and such a finite set of possible universes, how likely is it that there exists a life-sustaining universe?

16 The multiverse arguments that reject the Contingency Assumption also focus on the existence of our universe, but, for them, this is equivalent to certain laws/initial conditions being instantiated.

17 Everettian ‘many worlds’ theories are also very popular, but less appealed to as an explanation of fine-tuning. Having said that, there are some attempts to deal with fine-tuning via Everettianism (Wilson, 2020). It’s an interesting question whether such views would avoid the IGFC, but one outside the scope of this paper.

18 Although White disputes that the fine-tuning is evidence for the multiverse, he accepts that if we assume—perhaps on independent evidence—that there is a multiverse (of the right kind), the fine-tuning argument is undermined. Section VII of White (2000) explains how these can both be true.

19 Manson (2020) surveys cosmologists on questions relevant to this issue. In so far as the cosmologists took a stand on the Contingency Assumption, they seemed to be significantly more in favour of it than against. However, in more or less equal measure they found the phrases used to ask the questions—such as ‘our universe’ or ‘this very universe’—obscure. This is important and interesting data. However, it doesn’t obviously settle the issue, as in general physicists have neither been trained to address nor had experience in addressing these kinds of metaphysical questions.

20 Manson and Thrush (2003, p. 77) say that this kind of multiverse raises the question of what determines the identity conditions of regions in the larger space. It doesn’t seem to me that we need to answer this question in order to have grounds for holding that the identity conditions of the bubbles are determined by their location in the larger space. Moreover, either an account in terms of the relational properties of a given region, or one involving the postulation of haecceities at regions or points, would seem to be adequate.

21 One option would be to adopt analogous strategies to those endurantists adopt to avoid the problem of temporary intrinsics (Haslanger 1989; Lewis, 1986, pp. 202–204; Macbride, 2001). For example, one could hold that a single universe instantiates all of its properties not simpliciter but relative to a location in the multiverse. This would allow a single universe to be multiply located in the style of a Catholic saint. But one would need motivation for introducing all this metaphysical baggage, and the motivation shouldn’t be avoiding the IGFC. One should rather work out the most plausible version of a given multiverse hypothesis and then assess whether or not the Essentialist Response succeeds, relative to that hypothesis.
